# The Insanity of Men of Genius

**Published:** 1849-04-01

**Authors:** 


ART. IV.—*Torquato Tasso—Crichton Institution Biographies; or, Memoirs of Mad Philosophers, Mad Kings,* &c. No. 1. Dumfries, 1849

A GREAT—we were on the eve of saying an inspired—poet, has pointed out the close relationship existing between genius and insanity. We will not quote the apophthegm; it is to be found in every copy-book. Philosophy, history, physiology, psychology, and pathology, attest the truth of the observation. Who will presume to draw the line of demarcation between the highly wrought and gifted mind and the disordered intellect? Who is capable of distinguishing between the creations of genius and the wanderings of insanity? We dare not venture with Aristotle to state that it is essential for a good poet to be mad; but so much we will maintain, that the development of brain and nervous system on which the poetical temperament depends, is more nearly allied to that cerebral condition so often associated with insanity, than many are disposed to believe. That excessive expansion of nervous matter—great sensibility—acute sensitiveness—quickness of apprehension—and vividness of imagination, &c., are all indications of a state of brain bordering closely on the confines of disease, who can doubt?

The attempts which psychologists have made to define insanity have all signally failed. That there is in this condition of mind a want of balance between the representative faculty and the judgment, is clear; in such wise we may presume to define the visions of slumber-dreams, which may be said to differ from insanity *only* in degree or in duration.

It is true that slight shades of this want of balance may be daily witnessed in our intercourse with men; may we not therefore conclude that madness is a question of *degree*, and not of *kind*, and so cut the Gordian knot, or adopt in despair the wise saw of old Polonius, "to define true madness, what is it but to be nothing else but mad?"

The truth is this: the errors or contrasts of our definitions do not so much consist in our notions of insanity, as in our dilemma in drawing the hair-line of demarcation between sanity and insanity. If we could contemplate the *progress* of aberration in a mind, from simple abstraction down to drivelling idiocy, or to

> "Moody madness, laughing wild,
> Amid severest woe,"

where should we draw this line? Will the rules of psychology determine the question, and help us out of our dilemma? Or, on the conviction that there is imperfection in the most perfect mind, must we confess that there is none sane but the Deity, and content ourselves with quoting Gregory—"*Nulla datur linea accurata inter sanam mentem et vesaniam*"?

If a true or accepted definition of madness is so difficult, the *manifestations* of insanity are no less vague and uncertain, especially in the expression or action of that form termed *monomania*. How often have we known the acuteness or tact of a patient, on whom a jury were sitting, completely hoodwink these twenty-four wise men—ay, and eke the three learned Thebans in commission—until the string was touched which was "jangled and out of tune," and then the *mens insana* was as clear as the noonday sun!

A case is recorded of a gentleman of large possessions, who was convinced that Queen Charlotte of England was enamoured of him. His family, to induce the chancellor to grant a commission of lunacy, invited him to dine in company with the monomaniac. But the chancellor was deceived by the brilliant witticisms and conversational powers of the patient, who was indeed the life of the party, so that the learned judge congratulated him on his power of mind, and expressed his surprise at having been informed that the gentleman believed the Queen in love with him. "What!" exclaimed the lunatic

"am I to be considered crazy for giving credit to her most ample and repeated avowal of that being the case?" The commission was directly granted. Lord Erskine has related two similar cases. In both, the most rigid examinations had been tried in vain to establish insanity, until the one was accosted as the Saviour of the world, and the other was reminded of his amour with a princess, with whom he corresponded in cherry-juice, when the gentlemen instantly gave unequivocal evidence of insanity. A fine young officer, in whose case we attended a commission at Rochester, some years ago, had almost disproved our opinions by his rational answers, until, as he was retiring, he took a paper with great solemnity from his pocket, and read, "Home, sweet home," which he assured the jury *he had that morning composed* for their entertainment. We may add, also, that a gentleman committed suicide by dividing the brachial artery, after having passed the morning in professional visits, during which he evinced the most perfect judgment and self-possession. His illusion was the notion that his bones were decaying from the poison of syphilis. On this he cut his artery—but the evidence of the patients, without our own, would have established *felo de se.* Now, we may not be very far wide of the mark, if we affirm that every mind is prone to stamp those which think, speak, or act differently from itself, with the stigma of aberration: an hour scarcely passes in extensive association, without our hearing the expression, 'what folly!'—'why the man is mad!' &c., &c. This clearly evinces the estimation in which we are held by our fellow-beings who pride themselves on being, in Hamlet's phrase, "infinite in reason."

If these opinions are so constantly formed of the common herd of mankind, the 'profanum vulgus,' how much more closely may they apply to those,

> "Whose minds are dolphin-like, and soar
> Above the elements they live in"—

the children of Genius.

The thoughts and expressions of these gifted beings are essentially eccentric in the truest sense of the word, and therefore are they occasionally worshipped or reviled—contemplated with wonder, o regarded with pity. Like Beattie's precocious boy,

> "Some think them wondrous wise, and some believe them mad."

That deeply interesting, we may write *sublime* subject, in which we have engaged—the *insanity of genius,* itself a psychological problem, comes before us with the most awful contrasts: a spectral "life in death." "The light that leads astray, is light from heaven," and while we contemplate these Rembrandt shadows of the mind—this chiaroscuro of the soul of man, we at once confess their salutary but painful influence in curbing the pride of the heart, and teaching mankind, in most miraculous language, a practical lesson of humility. We may learn, even in this, that the wisdom of the Creator has ordained in animate nature something like a balance of happiness. We know excess of pleasure is succeeded by depression, while the apathetic moves on through life in one cool, monotonous, yet contented course. So has the light of learning, its shadow. Even Bacon's almost divine mind was not unalloyed with debasement.

In intellect, we scarcely read of one child of genius who has not sooner or later displayed the ravages which the struggle and labour for his fame, have worked in the organ and manifestations of his mighty mind.

In commenting on the aberrations of intellect, we must trace the malady to its source—to that degree or stage which *is* not yet, but which *may be* madness; for insanity, like other diseases, has its incubation and its development.

The very infancy of genius is often marked by such eccentric courses; as Samuel Johnson would almost term madness. He writes—"Madness itself merely discovers itself by unnecessary deviations from the usual modes of the world." Even enthusiasm has been stigmatized as insanity. Richardson termed Michael Angelo the "divine madman," and Oliver Goldsmith was designated "an inspired idiot." The eccentric pencil of our own Fuseli traced conceptions so out of the pale of common life, that we have often overheard very learned comments on the vagaries of mad Fuseli; and Turner, who produced such magical effect with gamboge, vermillion, white lead, and verdigris, is annually arraigned as a fit subject for a keeper, by the wiseacres in the rooms of the Royal Academy.

This eccentricity of genius often betrays itself by mere abstraction —a sort of *brown study*—in which the mind is so absorbed with the intensity of its creations, that impression of a sense does not impart such impression to the sensorium. The current stories of Pliny, Archimedes, Parmegiano, Marino, Newton, are very interesting illustrations of this *passive* eccentricity—this reverie of genius.

The vagaries of *active* eccentricity are displayed in the anecdotes of Hogarth, Geo. Harvest, and Sir Isaac Newton, with a degree of terseness, certainly, which savours somewhat of exaggeration; for if these sons of genius were so utterly regardless of their persons, how could they guard their purses? Still we know quite enough to be sensible of the absence of the common modes and courtesies of life in many of those whose works will live in glory, long, long after their eccentricities are forgotten.

These eccentric causes are sometimes developed even in infancy, and they are often the halcyons of intellect. But they do in reality also indicate a predisposition to aberration, if undue excitements be administered to or encouraged in the ill-fated subject of precocity. We cannot peruse the letters of Lord Dudley, one of those unhappy spirits who was *never a child,* to the Bishop of Llandaff, without a very sorrowing sympathy with his fate, and regret for the errors of his tutors, in forcing the germ of his intellect, and, as it were, wearing out his brain. We may look with pity too on many other more brilliant stars of literature, endowed with the so often fatal gift of genius, in whom intellect not only began to grow, but burst forth into blossom while yet its organ was in the bud. In some, vitality was protracted somewhat beyond the half century, but melancholy had marked them for its own long before their earthly course was finished. Such were Ariosto, and Dante, and Tasso, and Alfieri, and Pope, and Collins, and Cowper, and Swift. Others, in whom sensibility was in excess, and the organ of their intellect of more attenuated mould, "burned out quickly." Such were Burns, and Keats, and unhappy White—

> "Like sunbeam which on billow cast,
> That glances, but it dies"—

and Byron, who, in the "English Bards," wrote that beautiful apostrophe on White's early fate.

We cannot wonder that *illusion* should form one prominent characteristic of genius, inasmuch as the workings of the imaginative mind are but one continued and protracted course of ideal creation, ere the contemplation of nature and reality be well commenced, and judgment and comparison are matured by study, and the reading of the minds of others. Of this illusion, however, *when the mind is conscious,* it does not constitute insanity, any more than it can be termed disease, for the wandering may be under the control of the will, and the systematic functions and their organs also in a state of perfect integrity.

Our immortal Shakspeare has argued that the lunatic, the lover, and the poet

> "Are of imagination all compact."

This seeming poetic licence has been sometimes proved to be a truth. Nat. Lee illustrated the three qualities in his own person. His poesy was highly eulogized by Addison; but if we read the thoughts even of one of his lovesick girls, we shall see that his passion must have been that of a mad lover. Then while he was confined in his cell in Old Bedlam, we are told, a cloud passed over the moon, by the light of which he was writing the scene of a play, when he cried out, "Jove, snuff the moon." With all this, Lee seemed to have well remembered the *living* pictures around him, for this is his faithful portraiture of madness, in his "Cæsar Borgia:"

> "Like a poor lunatic that makes his moan,
> And for awhile beguiles his lookers on.
> His eyes their wildness lose,
> He vows the keepers his wrong'd sense abuse;
> But if you hit the cause that hurts his brain,
> Then his teeth gnash, he foams, he shakes his chain,
> His eyeballs roll, and he is mad again."

"The poet's eye in its fine frenzy rolling," "bodying forth the form of things unknown," is often thus closely illustrating the real insanity of genius, the simplest form of which is the *unconscious* creation of false ideas, and an absolute dominion of the representative faculty over the judgment.

This consciousness, then, may form the very link between poesy and madness—a seeming analogy, though really diametrical contrast—the one, the inspiration of a poet's reverie, the other, an ever-during phantom.

Thus the hallucinations of Tasso differed from the conscious creations of Shakspeare—the ideal revelations of Blake, (which were unfolded to us by the late John Varley,) from the wild eccentricities of Fuseli. Yet we doubt not that Shakspeare and Fuseli saw in their mind's eye as perfect an Eidolon as Tasso and Blake, but they *knew them* to be phantoms.

We will, in illustration, record the contrasted visions of Tasso and Tartini, the one related from Hoole's Life of Tasso, and the other by the accomplished maestro himself.

"At Bisaccio, Manso had an opportunity of examining the singular effects of Tasso's *melancholy*, and often disputed with him concerning a familiar spirit, which he pretended conversed with him. Manso endeavoured in vain to persuade his friend that the whole was the illusion of a disturbed imagination; but the latter was strenuous in maintaining the reality of what he asserted, and to convince Manso, desired him to be present at one of the mysterious conversations. Manso had the complaisance to meet him next day, and while they were engaged in discourse, on a sudden he observed that Tasso kept his eyes fixed on a window, and remained in a manner immoveable; he called him by his name, but received no answer; at last Tasso cried out, 'There is the friendly spirit that is come to converse with me; look, and you will be convinced of the truth of all that I have said.'

"Manso heard him with surprise; he looked, but saw nothing, except the sunbeams darting through the window; he cast his eyes all over the room, but could perceive nothing, and was just going to ask where the pretended spirit was, when he heard Tasso speak with great earnestness, sometimes putting questions to the spirit, sometimes giving answers: delivering the whole in such a pleasing manner, and in such elevated expressions, that he listened with admiration, and had not the least inclination to interrupt him. At last, the uncommon conversation ended with the departure of the spirit, as appeared by Tasso's words, who, turning to Manso, asked him if his doubts were removed. Manso was more amazed than ever; he scarce knew what to think of his friend's situation, and waved any further conversation on the subject."

Such was the *hallucination* of Tasso. This is the *reverie* of Tartini:

"One night, it was in the year 1713, I dreamed that I had made over my soul to his Satanic majesty. Everything was done to my wish; the faithful menial anticipated my fondest wishes. Among other freaks, it came into my head to put the violin into his hands, for I was anxious to see whether he was capable of producing anything worth hearing upon it. Conceive my astonishment at his playing a sonata with such dexterity and grace, as to surpass whatever the imagination can conceive. I was so much delighted, enraptured, and entranced by his performance that I was unable to fetch another breath, and in this state *I awoke.* I jumped up and seized upon my instrument, in the hope of reproducing a portion at least of the unearthly harmonies I had heard in my dream. But all in vain: the music which I composed under the inspiration, I must admit the best I have ever written, and of right I have called it 'the Devil's Sonata;' but the falling off between that piece and the sonata which had laid such fast hold of my imagination, is so immense, that I would rather have broken my violin into a thousand fragments, and renounced music for good and all, than, had it been possible, have been robbed of the enjoyment which the remembrance afforded me."

The indulgence to excess in this sort of phantasy, or the abandonment to it as a professional study, is replete with peril. To the real world the visionary is an alien; to his adopted country, a denizen; he is an outlaw to the beings around him. In the end the brain becomes a chaos,—a wreck. We will glean a few sentences, in proof, from the record of a child of genius, to whom we have already alluded. "When a sitter came I looked at him attentively for half an hour, sketching from time to time on the canvas. I wanted no more. I put away my canvas and took another sitter. When I

wished to resume my first portrait, *I took the man, and set him in the chair*, where I saw him as distinctly as if he had been before me in his own proper person. I looked from time to time at the imaginary figure, then worked with my pencil; when I looked at the chair I saw the man. Gradually I began to lose the distinction between the imaginary figure and the real person, and sometimes disputed with sitters that they had been with me the day before. At last I was sure of it; and then—all is confusion. I recollect nothing more. I lost my senses—was thirty years in an asylum."

Here, then, we see a voluntary deposition of the judgment and faculty of comparison; they had lost their dominion over the representative faculty, and thus was an eccentric illusion converted into insanity.

Our late friend, Dr. Wigan, has recorded a case of this insane *phantasie*, in which the Eidōlon was a double of the Seer. It is that of a haunted man, and might, perhaps, detract somewhat from the originality of a popular scribbler of Christmas tales:—

"I knew a very intelligent and amiable man who had the power of placing before his eyes *himself*, and often laughed heartily at *his double*, who always seemed to laugh in his turn. This was long a subject of amusement and joke, but the result was lamentable. He became gradually convinced that he was haunted by himself, or (to violate grammar for the sake of clearly expressing his idea) his *self*. This other self would argue with him pertinaciously, and to his great mortification sometimes refute him, which, as he was very proud of his logical powers, humiliated him exceedingly. He was eccentric, but was never placed in confinement or subjected to the slightest restraint. At length, worn out by the annoyance, he deliberately resolved not to enter on another year of existence; paid all his debts, wrapped up in separate papers the amount of the weekly demands, waited, pistol in hand, the night of the 31st of December, and as the clock struck twelve, fired it into his mouth."

It is not here that we may discuss the question of unity, duality, or plurality of mind; we refer our readers to the recent works of Holland, Barlow, and Wigan, on that abstruse question of psychology. We cannot, however, on reflecting on the phenomena of hallucination of genius, believe that mind, as it is manifested to us by its organ, is one indivisible essence, which itself creates an illusion, and at the same time informs us that it is so: this would almost imply that the brain as a unity was at the same time sound and unsound. But if we can believe that one cerebral organ, or one cerebral hemisphere, may be perfect, and the other or the rest imperfect, distorted, or diseased, the dilemma at once vanishes.

We know, we have seen that this partial disorganization, either from extreme congestion or change of tissues, has been accompanied by evidences of insanity, and very interesting analogies might be adduced to prove this. We take the case of a philosopher of extreme energy and deep learning, in whom this hemispherical disorganization of brain was discovered.

Dr. Wollaston had long been sensible of the etiology and prognosis of his case, the onset of which was declared by paralysis of the finger. Dr. Holland thus writes: "He was accustomed to take exact note of the changes, progressively occurring in his sensations, memory, and voluntary power. He made daily experiments to ascertain their amount, and described the results in a manner which can never be forgotten by those who heard him. It was a mind unimpaired in its higher parts, watching over the physical phenomena of approaching death, and what well deserves note, watching over the progressive change in those functions which seem nearest to the line separating material from intellectual existence." With all this spirituality, however, there lurked some leaven of self-interest. It was on the very approach of death (as we are assured) that he divulged the platinum secret, and his calculations seemed to imply that at least his organ, or one of his organs of number, was not deranged. This homage to Mammon, at such a moment, might sanction the belief that the diseased hemisphere somewhat interfered with the spirituality or philosophy of the prevalent indication of his master mind.

In our discussion of this paradoxical question in psychology, we must remember that the brain is the *organ,* not the *gland,* of the mind. We do not say the diseased or congested hemisphere *secreted* false ideas, and the sound one created the faculties of judgment and comparison to correct them. We rest on this affirmation, that the mind cannot be manifested aright through the medium of diseased structure.

Now, in illustration, we will adduce the analogy of the senses. The soul is an essence, bright, pure, celestial: from its combination with the senses results intellect, which we may very justly term one great sense, or a combination of all.

We know that the senses require their own proper element ere they can render us conscious of nature. The eye cannot see unless there be light, nor the ear hear without special undulation, nor the tongue taste unless sapid substances be applied to its papillæ. But sight will not be perfect unless the eye be healthy or entire, and yet rays of pure light are still playing about its lenses, and penetrating to the inmost depth of its diaphanous tissues: so if the brain be diseased, can there be integrity of intellect,—can there be thought, reflection, judgment, comparison? No. Yet the soul is there: but its manifestation as the mind is perverted by its diseased organ, as the ray of bright light is dimmed or distorted by the diseased structure of the eye. Is it not easy, then, to draw this analogy? As the light is to the eye, so is the soul to the brain; light is the essence of vision—the soul is the essence, or light, of the brain, the sublime source of all those phenomena which, as an aggregate element, we call mind.

We will, in carrying on this analogy, liken the varied tissues of the brain to the summit of the giant alp, Mont Blanc, and we will assume that the rays of the sun are playing on its peaks; the rays which fall on the snow-clad cone are reflected back in the effulgence of their purity, but those which impinge on a granite rock, on *their* reflection present to the eye a dark, rugged, and misshapen surface. So, the blending of the immortal spirit with a healthy brain will be evidenced by the higher and nobler sentiments and faculties of our moral and intellectual nature; but if it is manifested through a brain, diseased or disorganised, in its totality, or in any of its numerous folds, then are its evidences displayed in the sentiment and actions of man, more or less clouded or debased.

These hallucinations of genius may assume two contrasted forms, which, if acute or protracted, become the light or shadow of the visionary's existence. *Cheromania*, or the illusion of bright visions, and *demonomania*, or belief in the reality of Satanic visitation.

The exact proximate cause of these illusions may long elude the research of the most anatomical pathologist; the predisposition may depend on idiosyncrasy or constitution, fostered by indulgence or habit. Very many of these visions may, however, be referred to certain conditions of the blood. Hyperoxygenation of this fluid, whether from inhalation of nitrous oxyde gas, or even pure atmospheric air, will elevate and enliven the feelings and ideas. An excess of carbon, whether from inhalation or narcotic influence, or confined atmosphere, will ever induce depression and a gloomy mood of mind. The essence of cheromania may also consist in hyperoxygenation of the blood; that of demonomania, in congestion or remora of dark blood in the vessels of the brain.

We may observe analogous effects of temperament and disposition in the contrasted diseases of melancholia and phthisis. In the fluids of the former, carbon predominates, in those of the latter, oxygen; it may be from rapid circulation, or even from the free admission of oxygen to the blood in consequence of abrasion. May we assume, as an example, that from such an influence was developed those almost angelic traits of character which flung so bright a halo over the transient existence of our boy king, Edward VI.

To the cheromaniac every vision is gilded by a *couleur de rose*—every voice is the whisper of a seraph.

When the angel appeared to Jacob Behmen, he long listened with rapture to a voice of celestial modulation. The illusion of Benvenuto Cellini was a lofty cheromania, still increased by a mind supremely inflated by self-esteem. Thus he records one of these, (a conversion of natural truth into phantasy): "This resplendent light is to be seen over my shadow till two o'clock in the afternoon, and it appears to the greatest advantage when the grass is moist with dew; it is, likewise, visible in the evening at sunset. This phenomenon I took notice of when I was at Paris, because the air is exceedingly clear in that climate, so that I could distinguish it there much plainer than in Italy, where the moists are much more frequent; but I can see it there and show it to others, though not to so much advantage as in France."

Petrarch was thus blessed with the Eidōlon of Laura in his visions. Among other most interesting illusions of genius we may remember those of Edward, Lord Herbert, of Cherbury, of Malbranche, and Descartes. The mind of Shelley, also, (whom Locke would have at once termed a madman, on his axiom, that insanity is excess of morbid imagination,) was constantly prone to illusions of deep and painful sentiment—demonomania: but sometimes, perhaps, from the influence of surrounding circumstances, he became a cheromaniac. Percy Bysshe, with whom we wandered in our childhood, when he was a young Etonian, was a proselyte to spectral realism, especially when excited by visionary studies. We will glance, as an instance, to one of his day dreams, recorded by his friend, Williams, when they were in the isolated paradise of St. Arengo. One evening, after tea, as they were wandering in the moonshine, he suddenly grasped Williams' arm, and exclaimed, "There it is again—there;" and, closer questioned, he declared he saw his lately deceased child, naked, arising from the sea, and then clap its little hands as if in an ecstasy of joy, and looking on him with the smiling countenance of a cherub.

"I have dreamed of a golden land," said Fuseli in a rapture, "and solicit in vain for the barge that is to carry me over." The demon of Socrates (to which even the Satanic epithet of

κακο δαιμον has been applied) was, in reality, a sort of brownie, although this spirit, as the sage himself confessed, twice withheld him from offering any defence on his trial. Of the implicit credence of Socrates in the preternatural reality of his demon, we have many curious stories. This is recorded in the "Banquet of Xenophon:" "One Timarchus, a noble Athenian, being at dinner in company with Socrates, he rose up to go away, which Socrates observing, bade him sit down again, 'for,' said he, 'the demon has just now given me the accustomed sign.' Some little time after, Timarchus offered again to be gone, and Socrates once more stopped him, saying, he had the same sign repeated to him. At length, when Socrates was earnest in discourse, and did not mind him, Timarchus stole away, and in a few minutes after committed a murder, for which, being carried to execution, his last words were, 'that he had come to that untimely end for not obeying the demon of Socrates.'"

Tasso and his spirit were still more congenial, for the inspired poet seemed to court and welcome his mysterious visitant.

But even religious monomania will sometimes assume the nature of a bright vision. That devout monomaniac, John Mason, of Water Stratford, as Grainger informs us in his biographical history of England, was impressed with the conviction that he was the true Elias who was ordained to announce the advent of the Redeemer, and that when this awful epoch arrived, the millennium was to be commenced at Stratford. This visionary parson conversed and acted rationally on all subjects but those which referred to revealed religion, when he directly became mad. He died under the belief that he had just before been visited by the Saviour of the world, and in a complete faith in the reality of his own Divine mission.

Among others, the *celestial* visions and visitations of Emanuel Swedenborg are conspicuous, especially as they have enticed so many proselytes as to have established a sect which bears his name. The flights of his sister traveller to the heavenly region, Theresa, were followed by her apotheosis, and the canonization of her bones. This accomplished saint was enchanted by the writings of St. Jerome, and devoted herself to a monastic life. This metamorphosis, in a delicate frame, warm heart, and vivid imagination, induced melancholy, which ended in a cataleptic fit. In this trance she saw visions, and in her ecstasies she was lifted by supernatural powers from the earth into the presence of Peter and Paul, and even of the Redeemer himself, who once took her cross from her hands, and changed it into four large precious diamonds, &c.

The phenomena of cheromania may persist without the slightest constitutional disturbance; still it should ever be regarded with jealousy, as rapid or increased cerebral circulation may in a moment be induced, and terminate in frenzy. Even the sudden onset of high spirits, unmarked by illusion, should not be altogether slighted. We have known cases, especially in which honour and fame have suddenly been conferred on genius, in which the cerebral excitement has been suddenly followed by extravasation or effusion. In common minds, we have such effect of *high spirits* often evinced. A lady whom we knew had been for some time heavy and lethargic, when, for an hour or two before she attended morning service, she expressed her delight at feeling so infinitely better than she had been: she had scarcely entered her pew, ere she dropped apoplectic, and instantly expired. It was probably this hyperoxygenation of the encephalic blood which caused the exalted feeling in the cases of periodical mania related by Willis, in which the patient anticipated his insanity with extreme pleasure, and that also recorded by Pinel. The first patient affirmed thus: "Everything appeared easy to me; no obstacles presented themselves either in theory or practice; my memory acquired, all of a sudden, a singular degree of perfection. In general, I have a great difficulty in finding rhythmical terminations; but on these occasions, I wrote verses with as great facility as prose." Pinel refers to a literary gentleman of but ordinary powers who, during his paroxysms soared far above his usual degree of intellect. "He declaimed on the subject of the revolution with all the force, the dignity, and purity of language that this very interesting subject would admit of."

And in what do these phenomena of cerebral excitement differ from the cheromania of the first stage of intoxication? In nothing, save the duration of paroxysms and their exciting causes. Hyper-oxygenation of brain-blood is the essence of them all.

But the continuous thought and brooding over ideas by the brain of genius, in most instances induces a contrasted condition, which shows us the other side of the picture. Remora of the blood is marked by signs resembling the second stage of intoxication. The false idea, or Eidōlon, is often not altogether without the influence of the judgment; but as this faculty is seldom so potent as to dispossess the mind of the *presence* of the phantom, even if it suggests the conviction of its unreality, that mind is for the time monomaniacal. Such was the mind of Rousseau, when in the company of his phantom, and Luther, while he conversed, without suspicion, with the demon in his study. It induces the penalty of

Frankenstein; it has raised a monster beyond its control; just on the principle of our often inducing a pain or action in a part, by concentrating the attention on the spot. So Spinello, during his deep study for his picture of the fallen angels, kept his mind so especially concentrated on the conception of Lucifer, that the *horrible* shadow of the arch-demon was constantly before his eyes. Of these intellectual demonomaniacs, all coming under our category, we have recorded many very interesting cases. Jurieu, as we are informed by Dr. Beddoes, locked himself in his study for the deep analysis of the Apocalypse. The intense thought and heated air soon induced distressing maladies; these ended in the horrible illusion that the Beast of Blasphemy, with ten heads, and ten horns, and ten crowns on his horns, was pent up in his body, and was preying on his vitals!

Our lamented friend, Dr. Johnson, told us of a gentleman of great science who conceived that he was honoured by the frequent visits of spectres. They were at first refined and elegant, both in manners and in conversation, which on one occasion assumed a witty turn, and quips and puns and satire were the order of the evening, so that he was charmed with his ghostly visitors. On a sudden, however, they changed into demoniac fiends, uttering expressions of the most degraded and unholy nature. Depletion was the cure of this phantasy.

The physician of an acute Scotch lawyer had long witnessed some secret horror embedded in the mind of his patient, which, indeed, at length proved fatal; and at last extorted the confession that a skeleton was ever glaring on him from the foot of his bed. The doctor once endeavoured to dispel the illusion by standing directly in the field of the vision, and was himself startled, when his patient declared he saw the phantom peering at him over his left shoulder.

Such, too, was the "martyr philosopher," in the "Diary of a Physician," who, just before his death, saw a black figure removing his books from his study, throwing his pens and ink into the fire, and folding up his telescope, as if they were now useless. This was but a memory-shadow of his own previous occupation.

The solemn figure which induced Mozart to write the "Requiem," which was first, indeed, chaunted over his own grave, was doubtless but a phantom of his own creation. Such, also, was the illusion of Nicolai, the bookseller of Berlin.

The case related by Dr. Abercrombie may be considered almost in proof of our position. A gentleman of high literary attainments was constantly annoyed by the intrusion of an old woman in a black bonnet. She was, however, of the filmy order of ghosts, as the lock of the door was seen through her. Believing that she had missed her way, he arose from his deep study, and courteously showed her the door, when she instantly vanished. The change of position, by altering the state of circulation of the brain-blood, no doubt dispelled the phantom.

Such are some of the illusions of genius. They *may be followed* by disorganization, by membranous adhesion, effusion, &c.; but these changes are not *essential*, as the most unyielding illusions may exist with integrity of systemic function and of general health. Of these, the two well-known and very interesting stories of John Tilney Matthews and Simon Browne, may be adduced as illustrations. The one believed that he was haunted by a gang of pneumatic chemists, close to London-wall, who incessantly tortured him with an *air-loom;* the other, that "he had lost his rational soul." In both, the monomania was persistent, yet they were in bodily health; and although one was needlessly confined, both perfectly harmless in society.

The etiology of monomania, and of its converse, "folie raisonante," is a subject of very deep interest, especially as they usually occur in contemplative or scientific minds. The questions might illuminate each other; as, in the one, there seems to be a mad point, in the other, a sane point in the brain. In the highly intellectual mind, we are presented with a dark spot, or phantom; in a maniacal brain, we may sometimes observe a lucid spot, from which may emanate one of the higher faculties of the intellect. Thus, Dr. Rush writes of a judge and a divine, both confessedly insane, but whose discriminating judgment and refined eloquence on the bench and in the pulpit were admirable. Some of the ablest articles in "Aikin's Biography" were written by an inmate of a lunatic asylum during his confinement. We are told, also, that some parts of one of our national establishments were constructed from the plans of one of its inmates. Cibber tells us, in his Life of Lee, "I have seen a ship of straw finely fabricated by a mad ship-builder; and the most lovely attitudes have been represented by a mad statuary, in his cell."

These questions are of high interest and importance, involving as they do the points of metaphysical psychology, disordered quality of blood, and duality of mind; but at present we must control our discussion and analysis on these subjects.

The Irritability of genius is the first link in that series of mental phenomena which we may more properly term disease, associated as it is with the distressing condition of morbid or excited sensation.

The constitution of the mind is often acutely sensitive, shrinking like a mimosa from the breath of criticism; when such a state is fed by the wear and tear of brain, the effect may be perilous and destructive. Even the common intercourse of life is a fret to such a mind; there are no thoughts or sentiments in common, and the converse is strained and painful. Then there may be no just appreciation of talent by unintellectual persons, and hence the petulance that poets and essayists have displayed—of which Oliver Goldsmith and poor Chatterton may be apt examples. Seneca, we remember, affirmed that genius could not be happy with many people, as such association would ruffle the surface of the mind.

If we glance at the statistics of the comparative mortality of genius, we may form some notion of the final effect of different studies and pursuits. At the extremities of the scale, we have the natural philosopher and the poet; the aggregate duration of the lives of the former may be stated to be 75, of the latter, 57. In the quiet contemplation of nature, the mind is usually under a calm and devout influence, which softens the acuteness of feeling. It is the study of God's book, a search after ineffable truth.

The mind of the astronomer especially, whose enraptured eye contemplates the "majestic roof fretted with golden fire," is carried far above the influence of human passion, and the collision of earth—and is not our mother earth a Moloch, by a thousand secret poisons sacrificing her own children? Herschel, Halley, and Newton were octogenarians.

But the labour of the poetic mind is a *creation.* To the Creator, a world, a universe, is but the work of a will, a wish, a fiat: to the creature, even the birth of a thought may be an overwhelming struggle, a convulsive pang of parturition. It is recorded of more than one poet, that they wrote their verses six times over; Alfieri writes, "all my tragedies have been composed three times." So careful was Virgil to revise and polish his poetry, that he compared himself to a bear that was constantly licking his cubs into shape. In some, however, we may observe such energy of mind, such firmness of brain, and such high moral temperament, as may come unscathed from the trial. Walter Scott was long enabled, with impunity, to write nine volumes in as many months, taking still his prominent position in society; and Johnson, in seven years, compiled his gigantic Lexicon, and wrote the Rambler, with other minor compositions, and went through his routine of society daily. We are told, too, that he wrote forty-eight pages of the "Life of Savage" in sixteen hours; and our esteemed friend and colleague, Dr. C——, the Johnson of medical literature, may be adduced as one of the literary wonders of the day. But even these labours may perhaps yield, in the sapping and mining of brain, to the slavery of periodical literature.

If we contemplate the catalogue of imaginative writers, we shall discover signs of an irritable temperament in almost every one but Scott; his mind was cast in the mould of virtue, and his religion was as simple as it was sincere. Then Scott possessed a genuine unsophisticated love of nature, and raised himself to be a lord of the soil. Nor was he morbidly ambitious of literary victory, or jealous of the rivalry of other poets; thus he quietly yielded the laurel to Byron, and doomed his prolific pen to the composition of his more humble, though universally popular, prose. But let us glance at the *genus irritabile*—Cowley—Tasso—Dryden—Alfieri—Voltaire—Rousseau—Smollett—Pope—Johnson—Collins—Cowper—Keats—Byron, and we must almost pity the penalties of mighty genius. "Paganini, too," as our friend Dr. Moore writes, "paid dearly for his consummate excellence. Speaking to a friend, he stated that he scarcely knew what sleep was; and his nerves were wrought to such almost preternatural acuteness, that harsh sounds often became torture to him. His passion for music he described as an all-absorbing, a consuming one; in fact, he looked as if no other life than that ethereal one of melody were circulating in his veins; but he added with a glow of triumph, kindling through deep sadness, 'Mais c'est un don du ciel.'"

The effect of rapt attention is often derangement of memory—of which we have curious instances on record. Such was the transient oblivion of the acute scholar, (related by Crichton,) who had been deeply absorbed in studies, and at length was unable to write even what his will dictated—or to utter the words he wished. Thus, for "50 dollars, being one half-year's rate," which he intended to write, he scribbled—"50 dollars through the salvation of Bra—"

These derangements of the sensitive mind assume a variety of phases, eliciting, indeed, many of the most discordant passions of the human heart. Literary jealousy, for instance, will sever the most congenial friendship—as between John and William Hunter, and Chaucer and Gower, and others of less fame. The irritability of the ardent yet fragile mind will soon wear it out, especially if it be the subject of satire or censure. It is affirmed that the "end of Stillingfleet was hastened by Locke's confutation of his metaphysics;" that the *Quarterly* killed John Keats; and neglect of his genius impelled poor Chatterton to rush madly into the presence of his Maker; and Tasso, as if he feared his spirit even would feel a lash, begged Cardinal Cynthio to burn his works after his death, *especially* his *Jerusalem!* Even the poem of the Æneid, which, though never finished, had cost Virgil the labour of 11 years, was condemned to be burned, in the last will of the poet of Mantua. Happily, instead of throwing it into the flames, Augustus delivered it to Virgil's literary friends.

This sensitiveness will be roused even by the consciousness of personal deformity. The curved spine and skeleton legs of Pope, whose life was one protracted malady, might, perhaps, have much increased his acute feeling of censure. It is known, too, that the vanity of Byron was more than once deeply wounded when he perceived the eyes of a companion fixed on his club-foot; and when, almost a child, he overheard Mary Chaworth say to her maid, "Do you think I can love that lame boy," he ran in the full speed of passion across the fields from Annesley to Newstead.

Madden affirms Byron's disorder to have been epilepsy; and indeed he had many signs of cerebro-spinal disorder, as twitchings, globus, &c. on strong emotion. He had himself some notion of this being hereditary, as he tells us he was "cradled in convulsion," and "subject to a kind of hysterical merriment." A man so constituted as Byron was, who could write, "the seal of death is the only seal of friendship," must have innately so gloomy a view of society as to be a constitutional discontent, which he allowed to grow up with his life, for he was too great for sarcasm or reproof. None could such a mind endure, but a kindred spirit—a revolving satellite—or a doating woman. This misanthropy rendered him discontented with all but sensuality, and thus, much of his poesy, brilliant and triumphant as it was, was written as a resource against ennui, that "actual despair and despondency," with which he every morning awoke.

The "Bride of Abydos" was composed to keep him from "going mad by eating his own heart." And again he says, "I feel a disrelish more powerful than indifference. If I rouse, it is into a fury. I presume I shall die like Swift—dying at top." Byron's life, like his works, was a sort of Rembrandt study; so dark is all around, that the light shines out with a lustrous magic; the splendid poetry of "Manfred" gilds even the mystification of Astarte's fate, and we scarce pause to inquire its nature—incest, self-immolation, or what?—in our raptures at its poetic beauty. In such a conflict of wild, mad passions passed Byron's eventful life.

A similar morbid sense of critical comments influenced the late Lord Dudley, whose social position somewhat resembled Byron's; but he felt consoled by contrast, as when he knew the Governor-General of Bengal, with a genius so superior to his own, was not saved from "errors, humiliation, the taunts of his enemies, and the reproaches of his own conscience," which he corrects to "disapprobation of himself." And again, "W. R., a fellow-sufferer, is coming to dine with me, and his gloom. . . . I look forward with satisfaction to the *tête-a-tête*." His irritable susceptibility of brain was encouraged in his boyhood; he was one of those who never was a child, and was petted to excess by friends who were proud of his precocity. Lord Dudley lived with his elders, and had no *playmates*. His organic malformation of brain (as we learn from the *Quarterly Review*) was rivetted by the system of his education, which embittered his whole existence, and buried his bright prospects in the darkness of solitude and *insanity*. Lord Dudley's malady was often a conscious illusion; for if mad, he *knew* he was so, as he writes, "I am *ashamed* of what I feel." But there were undoubtedly traits of monomania, as in that morbid regret he felt for the possession of his great fortune. He seemed to form a contrast between what he was by birth and wealth, with what he should be, and bewailed his want of power in using his riches as he conceived that a Christian and philanthropist should do. He concludes one of his sorrowful letters to the Bishop of Llandaff with this severe sentence, "The iron had entered into my soul deeper than before."

Robert Burns was another of these specimens of the irritability of mind, in whom, notwithstanding an occasional gleam of splendour, existence was a penalty; for his life, after the hey-day of his warm-blooded youth, was one course of intoxication; it was a sort of champagne vitality; for while his system endured it, his leisure moments were devoted to the worship of Bacchus and of Venus; he was long the slave either of the thyrsus or the cestus.

It has been affirmed that Burns was, from his leading strings, hypochondriac. He himself tells us his constitution was blasted *ab origine* "with *incurable* melancholy." Alas! how little did he endeavour to effect its cure, especially after he left farming, and became indolent, slothful, and a toper. We grant that, to decide the struggle aright, between animal and intellectual, is no slight task; but the *sensitive mind* of genius must gain such a victory, or it will in the end *go mad*—we will not qualify the term.

That Burns (like Byron) possessed an *hypothesis* of morality and even virtue is evinced in his writings; but the madness of passion, that physical love which will ever be lord of all, while it inspired those warm outbreathings of the heart, was far out of the control of virtue, and thus was that heart reduced to a tainted sepulchre. Yet without this deep and unholy passion, though Burns would have been a happier and a better man, the world would have been shorn of the wild poesy of his sentiments. The amorous eulogies of his Marys, Janes, and Nancys would never have been penned; but his life would not have ended amidst the regrets of the libertine and the delirium tremens of the drinker.

If the assertion of Reid be accepted, that every nervous disorder is a degree of insanity, this *erethism of the mind* must be regarded as no slight form of it. Yet this madness may be very transient, for although in all increased actions or functions there is ever vascular excitement, this may subside, or even long continue without structural change. Yet this mere erethism must be ever regarded with extreme jealousy and suspicion; it may be the *incubation* of severe madness; if there be, especially, cerebral constitution or development to predispose, this simple erethism may end in acute frenzy. It has been decided by Pinel, Andral, and others, that the hope of recovery from insanity diminishes in a direct ratio to its previous duration. It were a wise and a benevolent precept, therefore, even in early youth, to check and keep down the enthusiasm of genius. But even in leading strings the most watchful care may be required for its surety. The precocious Blaise Pascal, even in his nursery, began to trace mathematical figures on the wall, and however selfish we may be to rejoice that Pascal was unfettered, yet he was in after life in constant horror of a yawning gulf before him, and was, indeed, bound to his chair during study, to prevent him from leaping, like Curtius, into the chasm.

But when the mind of genius is in its noon-tide of power, where then the giant arm to stem the torrent? As well may we attempt to curb with a nod the wild war-horse at the brink of a precipice, as to set a bound to the onspring of genius. Galileo, even in his seventy-eighth year, "could not prevent his restless brain from grinding on." But few brains possess the endurance of that of the inventor of the telescope. Alas! even in the dawn of youth many blaze for awhile and then sink for ever!

And, perhaps, we may exclaim, happy they who are thus early gathered to their rest, who, like spring violets, die in the fulness of their odour, but whose bright effusions, had they lived, would have beamed upon a selfish world, that would little dream of the pangs of composition by which they were ushered into light! The birth of the brain is often what the obstetrician would term a "laborious labour." If these labours are oft repeated, the systemic energy is reduced, and the climax may be, according to idiosyncrasy, temperament, or texture of brain, melancholy, frenzy, or drivelling idiocy.

"Since the 'Essay on Truth' was printed in quarto," writes Beattie, "I have never dared to read it over. I durst not even read the sheets to see if there were any errors in the print, and was obliged to get a friend to do that office for me. These studies came in time to have dreadful effects upon my nervous system; and I cannot read what I then wrote without some degree of horror, because it recals to my mind the horrors that I have felt after passing a long evening in those severe studies."

The excitement even of a thought in soft and sensitive brains induces various grades of mental disorder, from simple headache to confirmed mania—an influence highly exemplified in the melancholy life of Collins.

Hypochondriasis, that—

> "Loathed melancholy,
> Of Cerberus and blackest midnight born,
> In Stygian cave forlorn,
> 'Mongst horrid shapes, and shrieks, and sights unholy"—

probably really consists of morbid sensibility of stomach, associated with a predisposed brain; for we believe that few who are *unconscious that they possess a stomach* are really melancholic. The effect of these two causes is usually debility; *the* cause of Sydenham: or if protracted, they may induce hyperæmia and inflammatory action; *the* cause or condition of Wilson Philip.

We are too apt to tell a patient that he must not *lean* to his malady; but it is as wise to tell the leopard to change his spots, or the Æthiop his skin, as thus to dispossess the melancholic fiend. The hypochondriac must feel so long as the condition lasts; and with far more propriety may we resort to peristaltic persuaders, whether ammonia, rhubarb, colocynth, brandy, old port, hair gloves, or exercise, the best of all, than to a moral lecture or a scolding.

We pass by the religious monomaniac, Robert Hall. The two most interesting instances of religious melancholy were Samuel Johnson and William Cowper, yet in both the disorder assumed an especial individuality. The iron frame of the one endured and overcame; the other, like the reed, bent down before it.

Johnson was, at one time, constantly in terror as he looked into futurity, through the jaundiced medium of his malady. He probably brought from remote regions awful truths by his telescopic mind, and then even magnified them afresh by the lenses of his morbid imagination.

He said, "he inherited a vile melancholy from his father, which made him mad all his life; at least, not sober." He often told Dr. Swinfen that he should go mad; and seemed, indeed, close upon it. This notion Swinfen unhappily encouraged. Brocklesby would have acted more wisely. At twenty, his malady assumed a decidedly religious form; at fifty, he became irritable; at sixty, the dread of death and futurity again came over him; and he told Dr. Adams he would have a leg off to recover his spirits. This thought was ever on his lip—"To die, and go we know not where!" Some years before he died, Mr. and Mrs. Thrale found him on his knees praying with a parson *that he should not go mad.* Yet how shallow was even now this impression, for a hearty squeeze of the hand, and an assurance that he looked better, made him directly happy. It is probable that this mood was much enhanced by morbid imagination; for when his system sank under disease, his terrors of futurity waned, and he died resigned.

That the system of Johnson was afflicted with a strumous taint is certain. He had large scars in his neck, the cicatrices of glandular suppurations; and, indeed, he was carried when a child to Kensington, to be touched by Queen Anne for the evil.

We may believe, then, that unhealthy blood was circulating in his brain. But Johnson had really himself to thank for much of his melancholy. His habit was to rise late, to sit long, to study hard, to write voluminously, to wash his stomach out with hot tea, to feed heartily and ravenously, like one of the feræ, scarcely speaking during his gastronomic absorption, merely uttering a growl now and then, while the veins on his forehead were dilated, and his face sweated copiously with his exertions. We might seek far and wide in the records of etiology ere we could light on so perfect a catalogue of the *exciting* causes of hypochondriasis. Indeed, the neglect of peptic precepts by the deep student or constant thinker is of very evil tendency. Even Byron, after eating, often exclaimed, "Oh, fool! I shall go mad!"

During this repletion, the upper circulation is impeded, and the brain therefore becomes congested—a state favoured also by the energy of the circulation being expended on the assimilating process of the stomach. But in deep study immediately following this state, the organ of intellect is forced, and then begins the struggle of circulation. If the brain *beats,* and the blood is withdrawn from the stomach, dyspepsia arises at once, and then reaction may ensue in the brain, which may in the end induce even a phrenitic condition in its varied degrees.

The religious melancholy of Cowper was the offspring of a weak brain, which teemed with despondent and hopeless feeling. Some slight ray of hope might, for a moment, break sometimes on his clouded mind, when he wrote, "Joy of heart is the best of all nervous medicines," and then, perchance, he penned "John Gilpin." But the theme of his melancholy was this: "Spring flowers come after winter's frost, but a soul once slain, lives no more." He was a being of extremes, as he himself confesses; he was a mimosa in his youth, afraid even of a shadow, as when he resigned his office of reading-clerk to the House of Lords, from a dread of his public duty.

Cowper, like many others of sensitive minds, unhappily then retired to a world of his own, with pet hares for his companions, and water-lilies, and *alcoves*, and *hovels*, on the banks of the Ouse, about one of the most sluggish and muddiest rivers in England; all this tending to increase his malady, especially as he had somewhat crotchety people to deal with. One of these was his Platonic flame, Mrs. Mary Unwin, whose jealousy induced Cowper to turn off Lady Austin, whose cheerful accomplishments had cast a halo of happiness around him; a second and a third were the well-meaning, yet unwise clergymen, one of whom set him to the composition of the Olney Hymns, while in a state of acute hypochondriasis! the other, Dr. Madan, believing him the subject of *special visitation*, went metaphysically to work, and by precept and lecturing made poor Cowper ten times worse than he was. No doubt the Rose of the Alhambra, or Annot Lyle, would have been, as they were to Philip of Spain, and Allan Macaulay, by far the best physicians. We must remember, however, that Cowper himself deemed every effort at physical explanation of his malady, profanation, and referred one of his sudden recoveries to the influence of special Providence. When he was looking down on Southampton Water, he writes—"I felt the weight of my misery taken off; my heart became light and joyful in a moment,—the Almighty fiat, not by a gradual dawning of peace, but from a flash, as it were, of His life-giving countenance!"

From these glimpses of the life of Cowper, we may gather enough to comprehend the nature of his insanity. His system also had signs of a strumous taint, though not so marked as those of Johnson, and his digestion was inefficient: there were two causes of debility. He was acutely sensitive of atmospheric changes, and on the sedgy banks of the Ouse, especially in January, when his paroxysms were most severe, vapour clouds would be constantly floating. Thus was the atmospheric electricity disturbed, which might influence the delicate brain of Cowper, and thus cast the demoniac shadows over all his thoughts and ideas. Each form of poetic fancy hence became a demon; and this, deepened by the working of a superstitious mind, not only doomed the poor poet to a mad-house, but impelled him with the desire to give himself up, before his appointed time, to that judgment, which he believed would pronounce sentence of condemnation.

The most painful and distressing aberration of mind is that of a false *religion ;* it is almost a burlesque of that high and solemn destiny which, in a healthy mind, ensures peaceful content and humble adoration; and this especially when the imaginative faculty of genius presumes to draw aside the veil, and develop the mysteries of the apocalypse, which must await some gigantic event or revolution ere the intellect of man can dare to interpret the symbols of his Creator. Such is the climax of the frenzy of Kan, a student of Leipsic, whose mind for some time revelled in the deep and holy mysteries of the Revelation; it is quoted from the record of Professor Gruner, of Jena :—

" On the 4th of August, 1779, the neighbours were much alarmed on hearing Kan abusing his father. Upon knocking at the door, he opened it, and allowed them to come in : the father lay on the floor weltering in his blood, murdered by his son, who had stabbed him in fifteen different places, and had cut his throat. Kan walked backwards and forwards to the window, agitated alternately by contrition, the consciousness of his crime, and ebullitions of insanity. At one moment, he accused himself of having committed so horrible and unpardonable an offence; at another, he denied his having murdered his father, saying it was an old Jew and a cheat whom he had killed."

This fretting canker of the soul also marked the life of poor Collins, whose existence was but one continued scene of mental agony :—

> " He pass'd in maddening pain life's fev'rish dream,
> While rays of genius only served to show
> The thickening horror, and exalt his woe."

One fertile source of aberration may be in painful contrast—the wounds of pride and vanity, the failure of that fame which genius has fondly believed would be his guerdon, or that he cannot realize his ideal paradise; then, lured by an *ignis fatuus,* he often flies from the society of men to solitude, which feeds the smouldering flame of his discontent, and, in the end, converts him into an amiable misanthrope—"Homo solus aut Deus aut demon." To this, tædium vitæ soon succeeds,—its climax, suicide. This was the course to fate pursued by the ingenious author of "Lacon," and, alas! by many others.

Haydon was another of those gifted beings who was maddened by neglect: he had chalked out to himself a course of study in his art which was unpopular, or unfashionable, and, unhappily, he was endowed with a consummate conceit of his own exalted powers. He first wondered at, and deplored the errors of the public taste in not admiring him; and then, subdued by neglect and want, penned some wild sentences, painfully illustrative of the mad agony of his mind, and died by his own hand. It is probable that the *etiology* of suicide may depend much on the acuteness of sensibility; Chatterton and Haydon destroyed themselves under its influence. Had the minds of many other sons of genius been equally sensitive, or had their sensitiveness not been constantly drowned in oblivion by excesses, perhaps they might have done the same.

There is sometimes, however, a sort of safety-valve unexpectedly opened; when genius hits on, or searches for, an illustration of his own condition, or is impelled to work out his feeling.

One hypochondriac was in some dilemma regarding the author from whom he was about to quote a passage on the subject of suicide, and in searching for the lines, forgot his pistol.

Kotzebue, once in a state of melancholy, contemplated suicide; but this mad impulse was diverted to his pen, and hence the composition of his "Misanthropy and Repentance," ("The Stranger.")

When we recollect even the transient effects of emotion during composition, how deeply do we sympathize with these victims of self-immolation. We may allude to the "paroxysms and floods of tears," and the "weeping and raving" of Alfieri; the "head burning and face flushing" of Buffon; the hysterical respiration, the "rapid fire colouring the face" of Madame Roland; the "violent tumult of nerves" of Metastasio; these paroxysms are but a degree of that furor which inspired the mad pythoness of Virgil.

> "Her colour changed, her face was not the same,
> And hollow groans from her deep spirit came.
> Her hair stood up; convulsive rage possessed
> Her trembling limbs and heaved her lab'ring breast."

Yet it was often during such paroxysms that the muse of Byron was most propitious; even in his tertian ague, when the fit was on him, and he was delirious, Count Guiccioli informs us he composed some excellent verses. As if frenzy was as essential to qualify for a command of the Muses, as an Elizabethan Admiral said it was for the command of a fleet. Well might Machiavel forbid princes to study deeply, if such be the results.

It is not only intense devotion to science and literature which leads to acute mental maladies. Those minds which are constantly engaged in the collisions and jealousies of a political arena, are oft seen to fail in the struggle. Pitt, Fox, Canning, died in the meridian of life—Liverpool became apoplectic and imbecile—Whitbread—Romilly—Londonderry—Calcraft—became their own executioners; not like the Roman stoic, glorifying in the commission of suicide, as an exalted virtue, but in the thraldom of a mind diseased—under the dominion of frenzy.

We have elsewhere commented on the lethal influence of black blood on the brain. In one of these sad cases, the neglect of this unhappy condition was proved; for the suicide, after opening his artery, was discovered in the act of stuffing the wound with a corner of a napkin; the hæmorrhage had partially relieved the frenzied excitement in the brain.

Dryden was more wise. Reflecting, we suppose, on the hyperæmic brain under deep study, he was wont to let blood, and in fact, go into brief training, ere he set himself to any great work. How strange a contrast to the custom of Ben Jonson, who, as Aubrey states, "would many times exceede in drinke. Canarie was his beloved liquor: then he would tumble home to bed; and when he had thoroughly perspired, then to study."

The acute malady of the brain, phrenitis or frenzy, in its varied degrees, is but an excess of those conditions on which we have been commenting. Its milder forms may gradually subside—in some brains it may induce exhaustion, in others, structural change.

Of this distressing malady, we may observe several phases. The signs of fever may be slight or severe. In many cases, we observe that exaltation of the senses, so constantly dependent on increased action or excitement of the brain—from its undue circulation of scarlet blood. Thus we have hyperæsthesia of the skin, and especially morbid acuteness of the sense of hearing, not only indicated by confused sound, as tinnitus aurium, but by the faculty of recognising very remote undulations. Dr. T. Thomson, while labouring under a febrile attack, could distinctly hear the conversation of persons in the basement of his house, although he lodged in one of its upper rooms. In some, life passes in a state of dreamy slumber. From Tissot, we glean the story of many a child of genius whose mind has been kept in this state of *coma vigil,* even for many months. Boerhave informs us that after a deep study, he did not sleep for a period of six weeks. Pope, after a similar absorption of mind, lay in a state of protracted mental prostration; and Smollett, in a condition of half-dreaming wakefulness for the period of half a year. Zimmerman, in his "Experience in Physic," thus records the apposite case of a Swiss student.

"A young gentleman gave himself wholly up to the intense study of metaphysics. In a short time he began to experience an inertness of mind, which he endeavoured to shake off by renewed efforts of application. This increased the complaint, and he redoubled his exertions. This kind of contest lasted six months, during which the disease increased so fast that both body and mind suffered from it. The health of the body was soon restored by proper remedies, but the mind and senses became gradually more and more impaired, until they at last were subjugated by a complete stupor. Without being blind he appeared not to see; without being deaf he seemed not to hear; without being dumb he did not speak. In other respects he slept, drank, ate without relish and without aversion, without asking to eat or without refusing to do so. He was deemed incurable, and all remedies were laid aside: this state continued a whole year. At the end of this time some one read a letter to him with a very loud voice; he was agitated, and emitted a murmuring complaint, and applied his hand to his ear. This was taken notice of, and the person read still louder. He then gave a cry, and exhibited signs of the most acute suffering. The experiment was again tried, and his hearing was re-established by pain. Every other sense was successively excited on a similar principle, and in proportion as he regained the use of them, the stupidity appeared to be diminished; but the prostration of strength which followed, and the pain he sustained, brought him to the brink of the grave. At last, nature, without the aid of medicine, gained a complete victory. He recovered his wonted powers, and is, at this day, one of our first philosophers."

Tissot (Sur la Santé de Gens des Lettres) thus writes: "Quiconque a pensé fortement une fois dans sa vie, a fait cette experience sur soi-même, et il n'y a point d'homme de lettres qui ne soit sorti plusieurs fois de son cabinet avec un violent mal de tête et beaucoup de chaleur dans cette partie," &c.

It is, indeed, melancholy to reflect on the intense and enduring labour of the brain-workers long after severe headach has warned them to lay down the pen. In bodily exercise we commonly obey the dictates of sensation; when fatigued, we rest or repose. But even where congestion has been induced in the brain, and a state of indirect debility has ensued, we allow—nay, we urge the brain to labour on to its own severe detriment and the derangement of every function of the body.

All this points most emphatically to the wisdom, nay, the duty, of periodicity, or alternation, of labour and repose. The *strong* muscle or brain may easily recover its suspended power or energy after a judiciously apportioned labour; like Antæus, it may spring up from its depression in all its pristine vigour. But the asthenia of the weak brain is not so simple a state; thus, while Johnson grappled with and conquered his malady, the efforts of Cowper and Collins ended in a settled melancholy. After the gigantic labours of Burton, the author of "The Anatomy of Melancholy" became the impressive and painful illustration of his own prolific subject, as we especially learn from the Latin epitaph on his monument in St. Friedeswiede's at Oxford. In some of our burlesque writers and actors is often witnessed this asthenia of mind, this contrast of grave and gay, in the closet and in the world. Poor Hood, whose facetiæ kept the reading classes in a giggle, was himself reserved and silent in society. Liston and Grimaldi were melancholy men; and the story of the French harlequin is, beyond all others, deeply interesting. His private moments were darkly shadowed by intense hypochondriasis, and he consulted a learned physician on his case. The doctor, after his investigation, told him that he knew of only one remedy for his disorder, and that was to see the tricks of Carlini on the stage. The patient instantly burst forth with this piteous exclamation, "Alas! Alas! *I am Carlini.*"

Depression, asthenia, apathy, even oblivion, must then follow excitement as darkness does light, and thus we so often have extremes of psychological manifestations. Great sinners become devout saints —Unitarians, if they change their creed, Papists—and hence the laborious intellect so often sinks into a state of abject idiocy, the lowest grade of intellectual being—that

> "Last scene of all,
> That ends this strange, eventful history,
> Sans teeth, sans eyes, sans taste, sans everything."

As the slightest and earliest form of senile imbecility is loss of memory, so also is the primal indication of waning intellect, as memory is a faculty which seems to require an entire integrity of brain for its play.

> "Bring me to the test, (says Hamlet,)
> And I the matter will re-word; which madness
> Would gambol from."

Thus, the suspicion of Walter Scott of his failing power was first excited by his complete forgetfulness, want of recognition, of one of his own songs, at Lord Ellesmere's, and even the Muses, who are the "daughters of Memory," forsook him. And yet how impressive, when all around was dark, the triumph of thoughts of heaven over those of earth—may we write, of his flitting soul over his intellect—his genius was gone for ever, but the soul, ere it parted, whispered some words like these, as a parting sentiment to Lockhart:—"My dear, be good, be virtuous—that alone will make you happy here." And it were well if genius would take a hint from this dwindling of its creative power: yet Scott, though he confessed his last compositions "smelt of the apoplexy," still wrote on, each succeeding work more and more failing in its style and its conceptions. Byron, on the contrary, "burned out quickly," and therefore he died in the zenith of his literary glory.

In glancing at Scott's latter works, the pathologist may form a shrewd guess at the progress of that flaccid degeneration of tubular neurine, which probably began with his reverses, kept pace with his wondrous toil to liquidate his debts, and ended in imbecility. Yet the *early* memory of Scott was wonderful, and composition scarcely deserved the name of *effort.* It may perchance somewhat detract from his originality, but memory was the foundation pillar of his genius, so richly was his mind, like that of Samuel Johnson and John Hunter, stored with literary treasure.

The brains of Scott, and of him whom, though at a distance, he resembled more than any other in universality of genius, our own resplendent Shakspeare, would probably, under happier circumstances, have indicated a green old age; but Walter Scott was living, and William Shakspeare, although so few knew it, died in the meridian of his splendour, of a foolish excess, as we gather from the MS. Diary of Mr. Ward, of Stratford, who was intimate with Shakspeare's associates—the Lucys, the Cloptons, and the Coombes, and wrote his diary, now in the library of the Medical Society of London, some 40 years after Shakspeare's death. In this we read the following passage:—"Shakespear, Drayton, and Ben Jonson had a merry meeting, and, it seems, drank too hard, for Shakespear died of a feavour there contracted."

In the brains of imbecility resulting from mental labour, we almost always discover disorganisation. In that of Swift there were both ramollissement and effusion. Of the etiology, however, of this splendid lunatic, there are so many discrepant conjectures, that we forbear an analysis of his aberration. If, however, anything can teach us humility, it is to see the possessor of so fine a genius become in the end

"A driveller and a show."

If anything can prove, not only the divine nature of the immortal soul, it is its intimate and essential blending with an organ ere it can afford us the slightest manifestation of its existence or its reality: it is that so pure, so divine an essence, should *seem to be* in so abject a state when its organ is rendered unfit for its earthly abode. Who can believe that mind is the mere result, the consequence of organization, when he sees intellect so prostrate and debased? Who can doubt that it is the spirit *only*, as it speaks to us *through the brain*, when he sees the amiable Beattie thus express his dread: "A deep gloom hangs upon me and disables all my faculties, and thoughts so strange sometimes occur to me, as to make me fear that I am not, as Lear says, in my perfect mind;" or, especially, when he sees the feelings and the moods of the *immaterial* essence so changed in a few seconds by the repletion of a feast? Who can believe, also, that the soul itself and not its earthly organ is diseased, when reflection must directly whisper him that he is thus sapping the very foundation of the Christian's creed?

No, the insanity of genius is one of the many awful proofs of immortality; the *unfettered* spirit, that through its organ once moved the lips and pen to speak or write the syllables which still delight mankind, is unchanged—unchangeable; but the phenomena which our senses perceive, both of intellect and madness, are the result of health or disease *in that structure*, by its emancipation from which, the intellectual, yet tainted mind, becomes the pure immortal soul.

